# The surgical resection of the primary tumor increases survival in patients with EGFR-mutant advanced non-small cell lung cancer: a tertiary center cohort study

**DOI:** 10.1038/s41598-022-22957-9

**Published:** 2022-12-29

**Authors:** Ying-Yuan Chen, Po-Lan Su, Wei-Li Huang, Chao-Chun Chang, Yi-Ting Yen, Chien-Chung Lin, Yau-Lin Tseng

**Affiliations:** 1 Institute of Clinical Medicine, College of Medicine, National Cheng Kung University, No.1, University Road, Tainan City, 701 Taiwan; 2 Department of Surgery, National Cheng Kung University Hospital, College of Medicine, National Cheng Kung University, No.138, Sheng Li Road, Tainan City, 704 Taiwan; 3 Department of Internal Medicine, National Cheng Kung University Hospital, College of Medicine, National Cheng Kung University, No.138, Sheng Li Road, Tainan City, 704 Taiwan; 4 Department of Biomedical Engineering, College of Engineering, National Cheng Kung University, No.1, University Road, Tainan City, 701 Taiwan; 5 Institute of Biochemistry and Molecular Biology, College of Medicine, National Cheng Kung University, No.1, University Road, Tainan City, 701 Taiwan

**Keywords:** Non-small-cell lung cancer, Targeted therapies

## Abstract

Tumor resection could increase treatment efficacy of epidermal growth factor receptor (EGFR)-tyrosine kinase inhibitors (TKI) in patients with advanced EGFR-mutant non-small cell lung cancer (NSCLC). This study aimed to retrospectively analyze patients with advanced EGFR-mutant NSCLC from a Taiwanese tertiary center and receiving EGFR-TKI treatment with or without tumor resection. A total of 349 patients were enrolled. After propensity score matching, 53 EGFR-TKI treated patients and 53 EGFR-TKI treated patients with tumor resection were analyzed. The tumor resection group showed improved progression-free survival (PFS) (52.0 vs. 9.8 months; hazard ratio [HR] = 0.19; p < 0.001) and overall survival (OS) (not reached vs. 30.6 months; HR = 0.14; p < 0.001) compared to the monotherapy group. In the subgroup analysis of patients with newly-diagnosed NSCLC, the tumor resection group showed longer PFS (52.0 vs. 9.9 months; HR = 0.14; p < 0.001) and OS (not reached vs. 32.6 months; HR = 0.12; p < 0.001) than the monotherapy group. In conclusion. the combination of EGFR-TKI and tumor resection provided better PFS and OS than EGFR-TKI alone, and patients who underwent tumor resection within six months had fewer co-existing genomic alterations and better PFS.

## Introduction

The epidermal growth factor receptor (EGFR) mutation is the most common oncogenic gene among patients with advanced-stage non-small cell lung cancer (NSCLC)^1^. Several phase III studies have demonstrated that the use of first- or second-generation EGFR-tyrosine kinase inhibitors (TKI) could increase progression-free survival (PFS) compared to platinum-based chemotherapy in advanced NSCLC patients with EGFR mutations^2–9^, which makes EGFR-TKI the mainstay treatment strategy for this condition. Studies focused on head-to-head comparison revealed that second-generation EGFR-TKI, afatinib and dacomitinib, show significant PFS improvement compared with first-generation EGFR-TKI^10,11^. Moreover, the phase III FLAURA study further demonstrated better PFS and overall survival (OS) than first-generation EGFR-TKIs. Although the FLAURA study showed promising results, the use of osimertinib, a third generation EGFR-TKI, as the first-line treatment remains controversial^12–14^. First, the GioTag study revealed that the sequential use of afatinib and osimertinib has a median OS of approximately four years in patients with acquired T790M resistance^15^. Moreover, there are no head-to-head comparisons between first-line osimertinib and sequential use of second-generation EGFR-TKI followed by osimertinib as second-line treatment. Second, the incremental cost-effectiveness ratio for osimertinib was higher than first and second-generation TKI in many studies^16–18^. Thus, sequential treatment could be an alternative, and the extension of PFS by the first-line treatment becomes an essential point of consideration.

The combination of EGFR-TKI and other treatments has been widely studied. A first promising treatment strategy is the combination of EGFR-TKI and vascular endothelial growth factor (VEGF) pathway inhibitors. In the phase III study NEJ026^19^, the combination of erlotinib and bevacizumab, an anti-VEGF monoclonal antibody, increased PFS and objective response rate (ORR) compared with erlotinib alone. Another phase III RELAY study^20^ also demonstrated the significant prolongation of PFS when combining erlotinib with ramucirumab, a VEGF receptor 2 monoclonal antibody. However, both these trials failed to demonstrate the benefit in OS. The second strategy was combining chemotherapy with EGFR-TKI. Both the phase III NEJ009 study in Japan^21^ and the phase III TATA study in India^22^ revealed that the combination of gefitinib and platinum-based chemotherapy increased PFS and OS compared with gefitinib alone. However, more than half of the patients in these two trials developed grade 3 toxicities, mainly hematological, which limited its application in clinical practice. Therefore, the surveillance of novel combination strategies is warranted.

Many studies have revealed that local ablative treatment can improve the treatment outcome in patients with advanced NSCLC who received chemotherapy^23,24^. Similar results were also found in patients with EGFR-mutant NSCLC. The application of consolidative local ablative treatment could significantly improve the PFS and OS among patients with EGFR-mutant NSCLC^25^. Another cohort study also demonstrated that local treatment to the site of progressive disease could prolong PFS and OS in EGFR-mutant advanced NSCLC patients with acquired resistance to first-line EGFR-TKI^26^. As a kind of local treatment, surgery was also studied to increase the efficacy of systemic treatment. A study analyzing the Surveillance, Epidemiology, and End Results (SEER)-registered database demonstrated that thoracic surgery could improve the prognosis in patients who received chemotherapy^27^. However, studies focused on the role of surgery among patients with EGFR-mutant NSCLC who received EGFR-TKI provided inconsistent results due to the heterogeneous patient population^28–30^. Recently, a retrospective cohort revealed that patients who underwent a resection of the primary tumor had a significantly better outcome than those who did not; however, most patients in the study had recurrence after curative surgery^31^.. In addition, whether surgery also has benefits among patients with newly diagnosed NSCLC remains unknown.

Our previous study documented that salvage pulmonary resection after TKI was safe and feasible^32^. In the current study, we performed a retrospective study with propensity score matching (PSM) analysis to overcome selection bias, increase the evidence level, and investigate the implementation of tumor resection in routine clinical practice for patients with EGFR mutations and advanced NSCLC treated with EGFR TKI.

## Materials and methods

### Patient population

From July 1st, 2013, to December 31st, 2020, all patients with newly diagnosed or recurrent EGFR-mutant advanced NSCLC who visited a hospital in southern Taiwan were enrolled in the study. All patients received complete staging examination including chest computed tomography (CT) scan, bone scan, and brain imaging [CT or magnetic resonance imaging (MRI)] based on the tumor, node, metastasis (TNM) system proposed by the American Joint Committee on Cancer, 7th edition. Patients who had stage I–IIIA or did not receive EGFR-TKI treatment were excluded. This study was approved by the Review Board and Ethics Committee of National Cheng Kung University Hospital (NCKUH B-ER-108-324). The baseline characteristics of these patients, including age, sex, mutation subtype, performance status, initial brain metastasis, and TNM staging, were recorded. The surgical resection of the primary tumor was performed at the discretion of the treating providers.

Given that all the patients who underwent primary tumor resection had a good performance status (ECOG 0–1), other patients with lower performance status (ECOG ≥ 2) were excluded. All data were anonymized, and, given the study’s retrospective nature, the need for written informed consent was waived by the Review Board and Ethics Committee of National Cheng Kung University Hospital (NCKUH B-ER-108-324). This research was carried out following approved guidelines and the Declaration of Helsinki. The residual tumor specimens of all patients who underwent surgery were evaluated by genomic testing. Adequate samples were tested for targeted sequencing of 409 cancer-related mutations by ACT Genomics (Taipei, Taiwan) with their next-generation sequencing platform ACTOnco panel^33^.

### Outcomes analysis

All the patients underwent computed tomography of the chest every 12 weeks after the initiation of EGFR-TKI treatment to evaluate their tumor responses. Brain imaging and bone scans were performed if related symptoms were present. The primary endpoint was progression-free survival (PFS), and the secondary endpoint was overall survival (OS). PFS was calculated from the date of EGFR-TKI initiation until the date of radiological progression according to the Response Evaluation Criteria in Solid Tumors (RECIST) v1.1^34^ or death, with censoring at the date of the last follow‐up if the patient had not progressed. The duration of OS was defined as the period from EGFR-TKI treatment initiation until death. Both the PFS and OS were followed up until December 2020.

### Statistical analysis

The frequencies and descriptive statistics of the demographic and clinical variables were calculated. Categorical variables were compared using the Chi-square test or Fisher’s exact test, whereas continuous variables were compared using Student’s t-test or the Wilcoxon rank-sum test. The PFS and OS were estimated by the Kaplan–Meier method and compared using the log-rank test. We also performed Cox proportional hazards regression for the predictors of PFS and OS. The selection of possible predictors and determinants was based on prior studies^35,36^. Age, sex, tumor size, nodal stage, EGFR mutation subtypes, and tumor resection were chosen as the predictors and prognostic factors. Subgroup analyses of PFS and OS were also performed by age, sex (male versus female), disease stage (newly diagnosed versus recurrence), brain metastases at baseline (presence versus absence), EGFR mutation type (exon 19 deletion versus Leu858Arg substitution). Statistical Analysis System^®^ software version 9.4 (SAS Institute, Cary, NC, USA) was used to perform the analyses. All the reported p values are two-sided.

We matched one patient who received EGFR-TKI and tumor resection with one patient who received EGFR-TKI alone (without replacement) by propensity score matching using the nearest-neighbor method based on the estimated propensity scores. Propensity scores were computed using logistic regression. Selected covariates included age (≥ 70 years vs. < 70 years to ≥ 60 years vs. < 60 years), sex (male vs. female), stage (recurrence vs. newly diagnosed), mutation subtype (Exon 19 deletion vs. Exon 21 L858R substitution), and presence of brain metastasis (presence vs. absence). The balance between patients who received surgery and propensity score-matched patients receiving EGFR-TKI alone was measured using standardized differences, expressed as percentages. An absolute value of < 10 suggests that the two groups are well balanced^37^. To account for the matched design, we also performed paired t-tests.

## Results

### Patient characteristics

**Figure 1:**
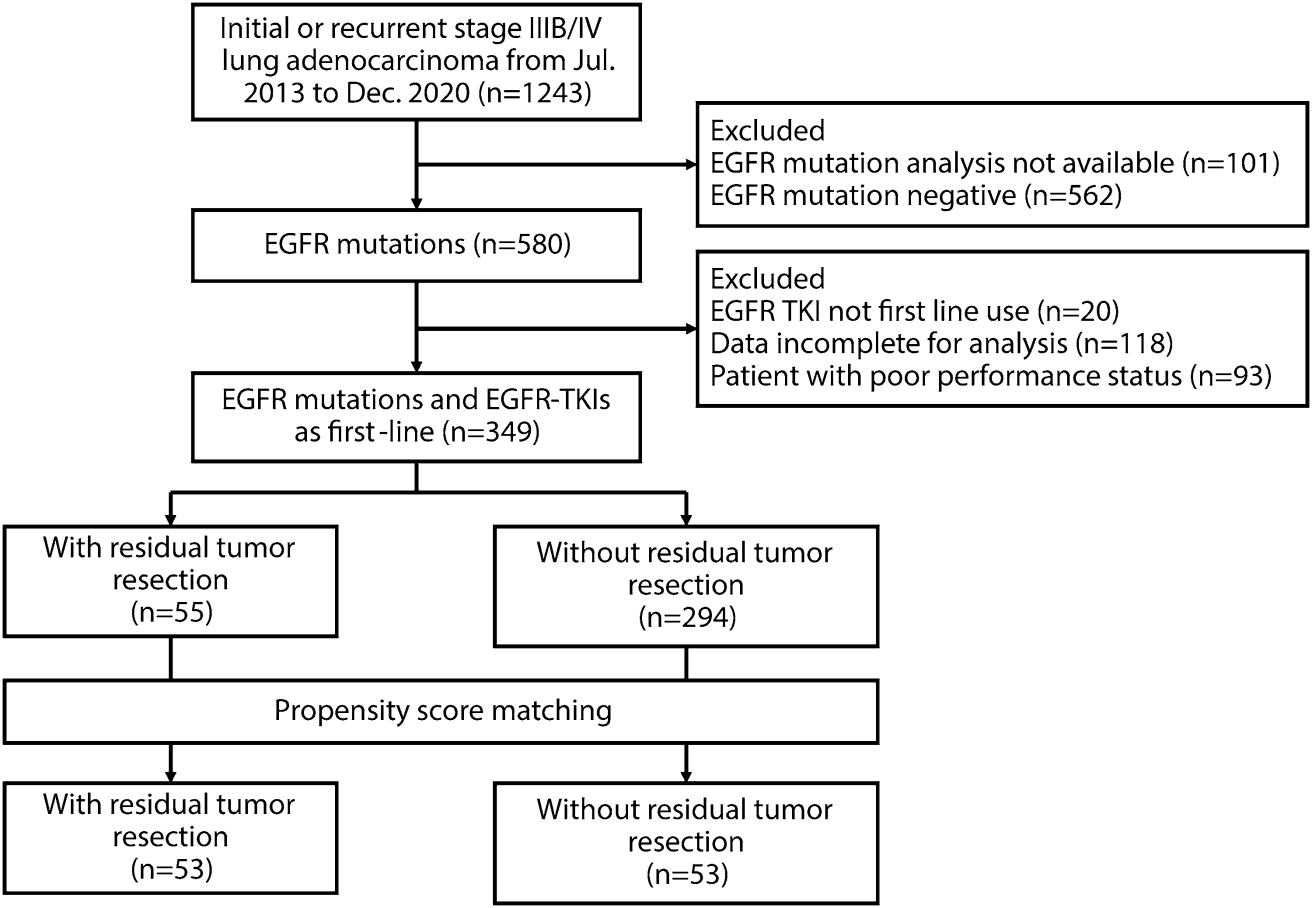
Flowchart for patient enrollment. *EGFR* epidermal growth factor receptor, *NSCLC* non‐small cell lung cancer, *TKI* tyrosine kinase inhibitor.

A total of 349 newly diagnosed or recurrent EGFR mutation-positive advanced NSCLC patients who visited the hospital from July 1st, 2013, to December 31st, 2020, were enrolled (Fig. 1). All enrolled patients received EGFR-TKI as first-line treatment, and 55 (15.8%) underwent surgical resection of the primary tumor, including 44 patients with tumor resection and partial EGFR-TKI response, three patients with primary tumor resection at the time of diagnosis, five patients with surgical resection for post-operative loco-regional recurrence, and three patients with surgery for loco-regional progression after EGFR-TKI use. Of the 44 patients who received surgery after partial response to EGFR-TKI, 16 patients received first-generation EGFR-TKI, and 28 patients received second-generation EGFR-TKI. The median time from EGFR-TKI initiation to tumor resection was 5.9 [3.0–9.3] and 4.3 [3.5–7.4] months in patients receiving first-generation and second-generation EGFR-TKI, respectively. After PSM, 53 EGFR-TKI-treated patients who received tumor resection and 53 patients with no tumor resection were analyzed. The baseline characteristics of the patients with and without surgery are summarized in Table 1. The demographic data of patients who were additionally treated with or without surgery were well-balanced in age, sex, performance status, stage, brain metastasis, and EGFR mutation subtype.

**Table 1:** Demographic and clinical characteristics of all patients.

	Primary tumor resection (N = 53)	No primary tumor resection (N = 53)	Standardized difference^a^
Age	64.6 [54.9–69.0]	62.5 [56.2–68.7]	
Age < 60 years	22	21	3.843
Age ≥ 60 and < 70 years	20	21	− 3.875
Age ≥ 70 years	11	11	0
**Sex, *n* (%)**			
Male	14	14	0
Female	39	39	0
**Stage**			
Recurrence	4	3	7.603
Newly diagnosed	49	50	− 7.603
**Brain metastasis**			
Presence	9	9	0
Absence	44	44	0
**EGFR mutation**			
Exon 19 deletion	20	20	0
L858R substitution	33	33	0

*EGFR* epidermal growth factor receptor P.

^a^Standardized difference (%) is the mean difference divided by the pooled standard deviation.

Among patients who received residual tumor resection, most of them had a primary tumor in the left upper lung (n = 16), followed by left lower lung (n = 14), right lower lung (n = 14), right upper lung (n = 6), right middle lung (n = 2), and multiple lobes (n = 1). Only two patients in the surgery group had tumor invasion to the mediastinum or chest wall. The remaining patients with T3 or T4 disease had separate tumor nodules in the ipsilateral lung. Before propensity score matching, patients with distant organ involvement, including brain, liver, bone, and adrenal gland, mostly received EGFR-TKI monotherapy (Supplementary Table 1). Although the difference became insignificant after propensity score matching, patients who did not receive residual tumor resection still had a marginally higher proportion of bone metastasis (p = 0.076, Supplementary Table 2). In contrast, the proportion of patients with mediastinal lymphadenopathy was similar between patients with and without residual tumor resection (Supplementary Table 2). Additionally, three patients in the surgery group had received radiotherapy during the use of EGFR-TKI. In contrast, only two patients in the monotherapy group had received brain irradiation. Fifteen patients received a segmentectomy only, and the remaining patients received a lobectomy. All patients underwent video-assisted thoracic surgery. Three patients had a microscopic residual tumor (R1 resection), whereas the remaining patients had complete resection (R0 resection). Forty-four patients in the surgery group had mediastinal lymphadenopathy at initial diagnosis; 29 of them had a pathological response in the lymph node (post-operative N0 disease) after EGFR-TKI therapy. Ten patients (23.8%) had a major pathological response, and one patient (2.4%) had a complete pathological response following tumor resection. Post-operative complications were minimal. The detailed data regarding the surgery group was summarized in Supplementary Tables 3 and 4.

### Survival outcomes of all patients

**Figure 2:**
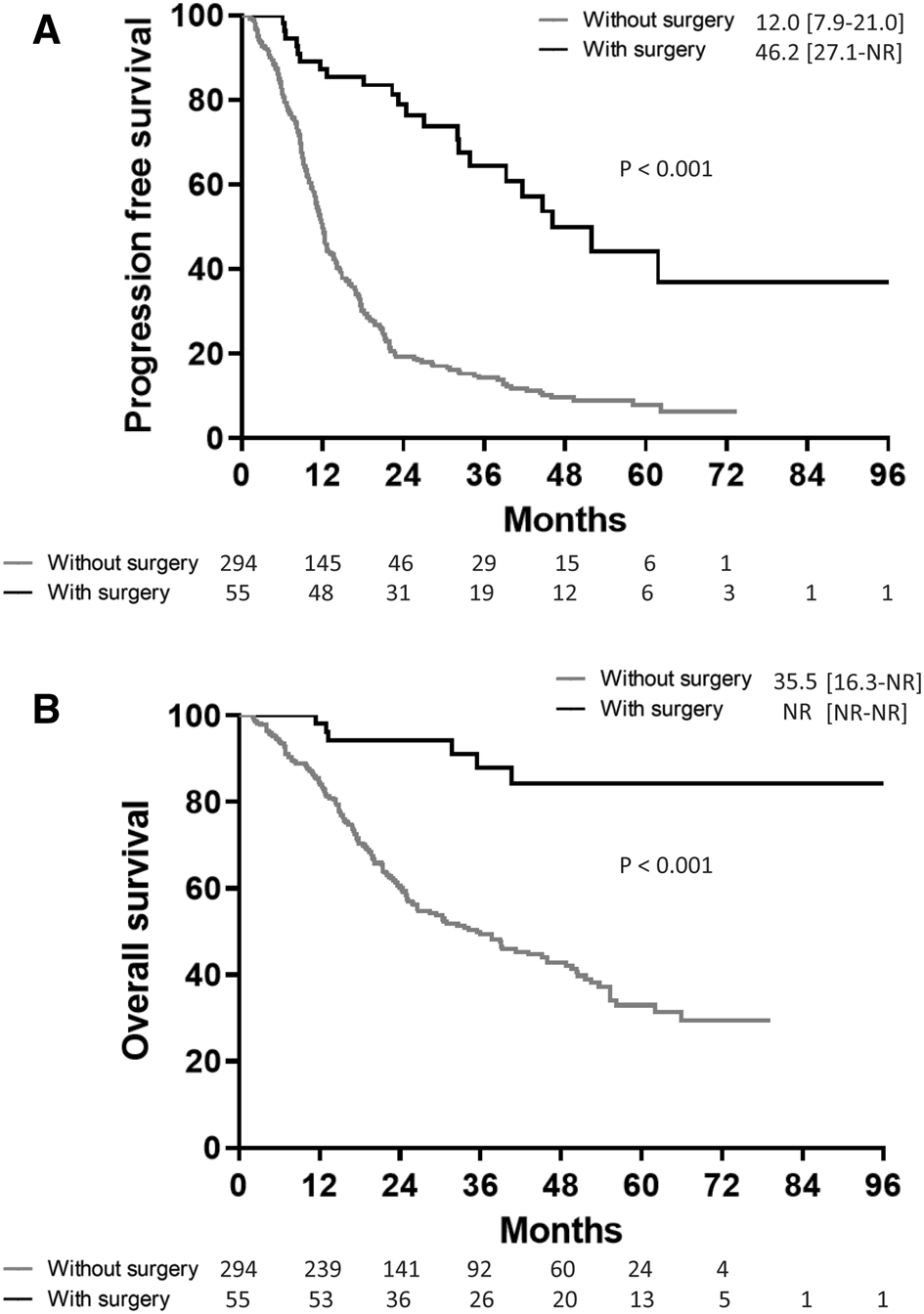
Progression-free survival (**A**) and overall survival (**B**) in epidermal growth factor receptor (EGFR) mutation-positive non-small cell lung cancer patients with and without primary tumor resection.

**Figure 3:**
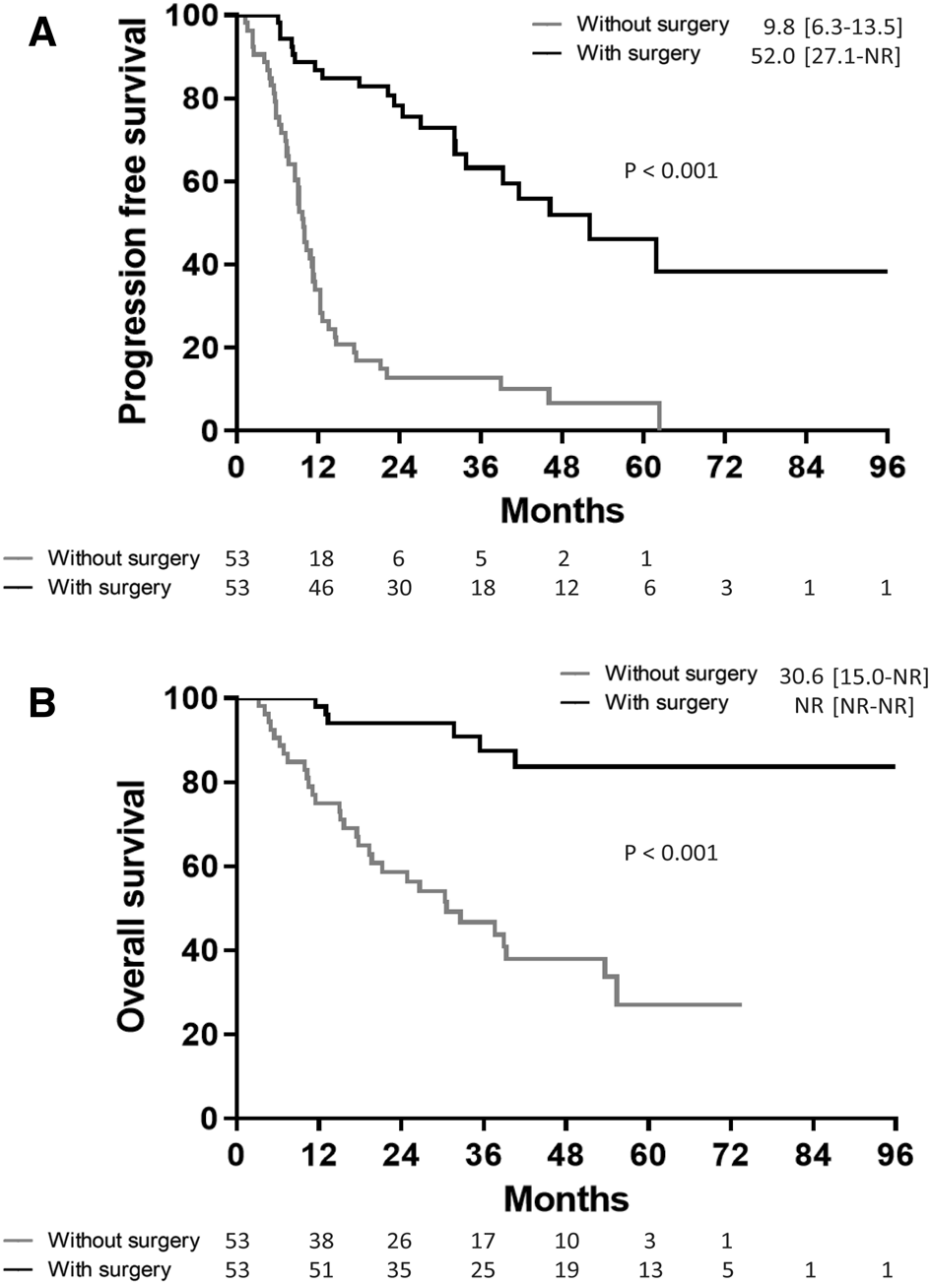
Progression-free survival (**A**) and overall survival (**B**) in epidermal growth factor receptor (EGFR) mutation-positive non-small cell lung cancer patients with and without primary tumor resection after propensity score matching.

Comparisons of PFS and OS between total patients receiving EGFR-TKI treatment with and without tumor resection were made (Fig. 2). The median PFS and OS in patients with tumor resection was demonstrated using Kaplan–Meier analysis to be significantly longer when compared to patients without tumor resection (log-rank test, p < 0.001 and p < 0.001, respectively; Fig. 2A,B). After propensity score matching was performed, the median PFS in patients with tumor resection was determined using Kaplan–Meier analysis and found to be 52.0 months [interquartile range 27.1–not reached (NR) months], which was significantly longer when compared to patients without tumor resection (log-rank test, p < 0.001; Fig. 3A). In addition, the median OS in patients with tumor resection was not reached, and demonstrated to be significantly longer when compared to patients without tumor resection (log-rank test, p < 0.001; Fig. 3B). Possible confounders were adjusted using Cox proportional hazards regression analysis, and the hazard ratios (HR) of PFS and OS for surgery were found to be 0.16 (95% confidence interval [CI] = 0.09–0.29, p < 0.001) and 0.14 (95% CI = 0.05–0.36, p < 0.001), respectively.

### Subgroup analysis

**Figure 4:**
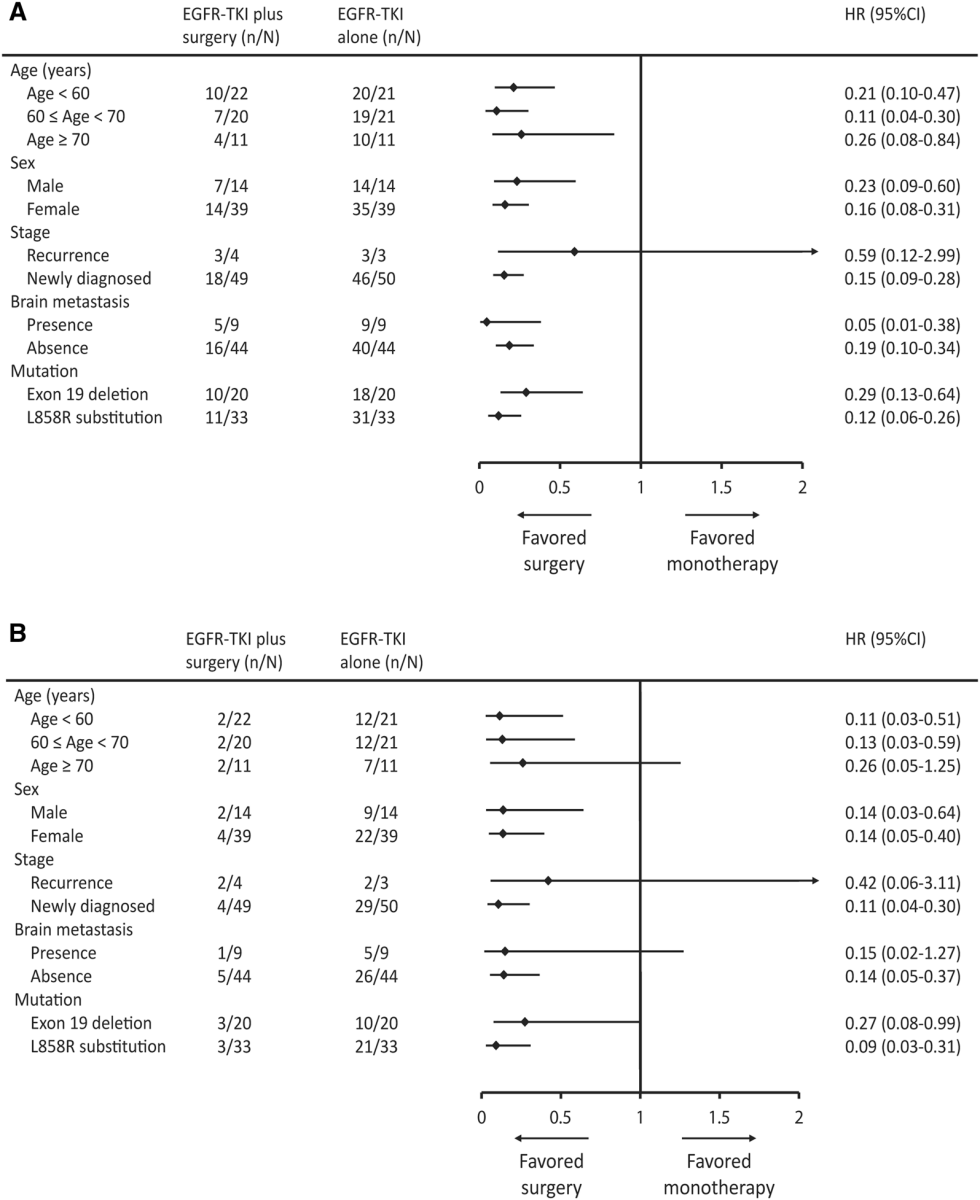
Subgroup analyses of progression-free survival (**A**) and overall survival (**B**) by baseline characteristics.

**Figure 5:**
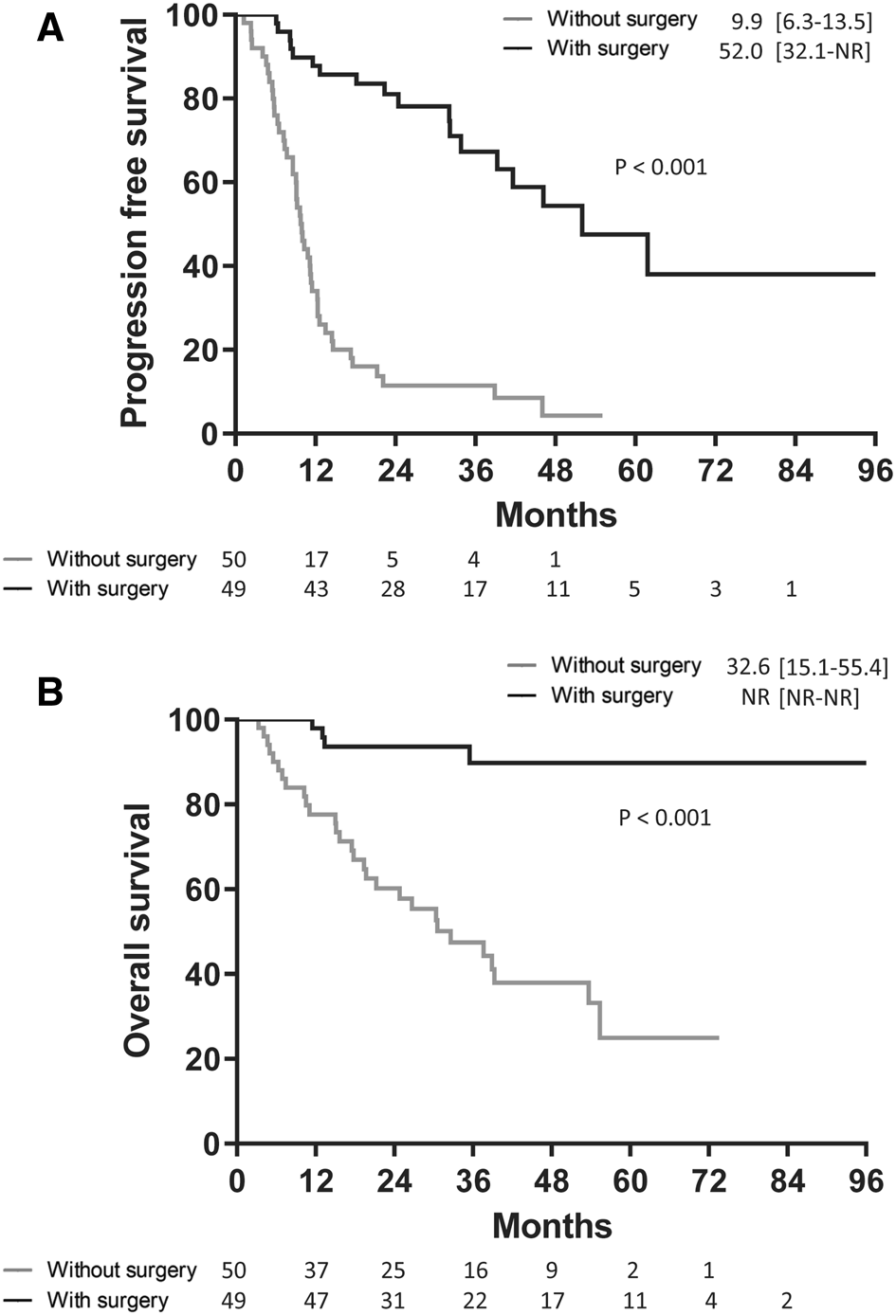
Progression-free survival (**A**) and overall survival (**B**) in patients with newly diagnosed EGFR mutation-positive non-small cell lung cancer with and without primary tumor resection.

A subgroup analysis based on patients’ characteristics demonstrated PFS HR and was in favor of tumor resection in most subgroups (Fig. 4A,B). Crucially, PFS and OS HR both favored surgery in patients with newly diagnosed NSCLC (Fig. 4A,B). In patients with newly diagnosed NSCLC, the median PFS was determined using Kaplan–Meier analysis and found to be 52.0 months [interquartile range 32.1–NR months] among patients receiving the tumor resection; this period was significantly longer when compared to patients without tumor resection (log-rank test, p < 0.001; Fig. 5A). Additionally, the median OS was not reached [interquartile range, NR–NR] in the tumor resection group; and this was significantly longer when compared to patients without tumor resection (log-rank test, p < 0.001; Fig. 5B). Possible confounders were adjusted using Cox proportional hazards regression analysis, and the HRs of PFS and OS for surgery were demonstrated to be 0.14 (95% CI 0.07–0.26, p < 0.001) and 0.12 (95% CI 0.04–0.36, p < 0.001), respectively (Table 3).

**Figure 6:**
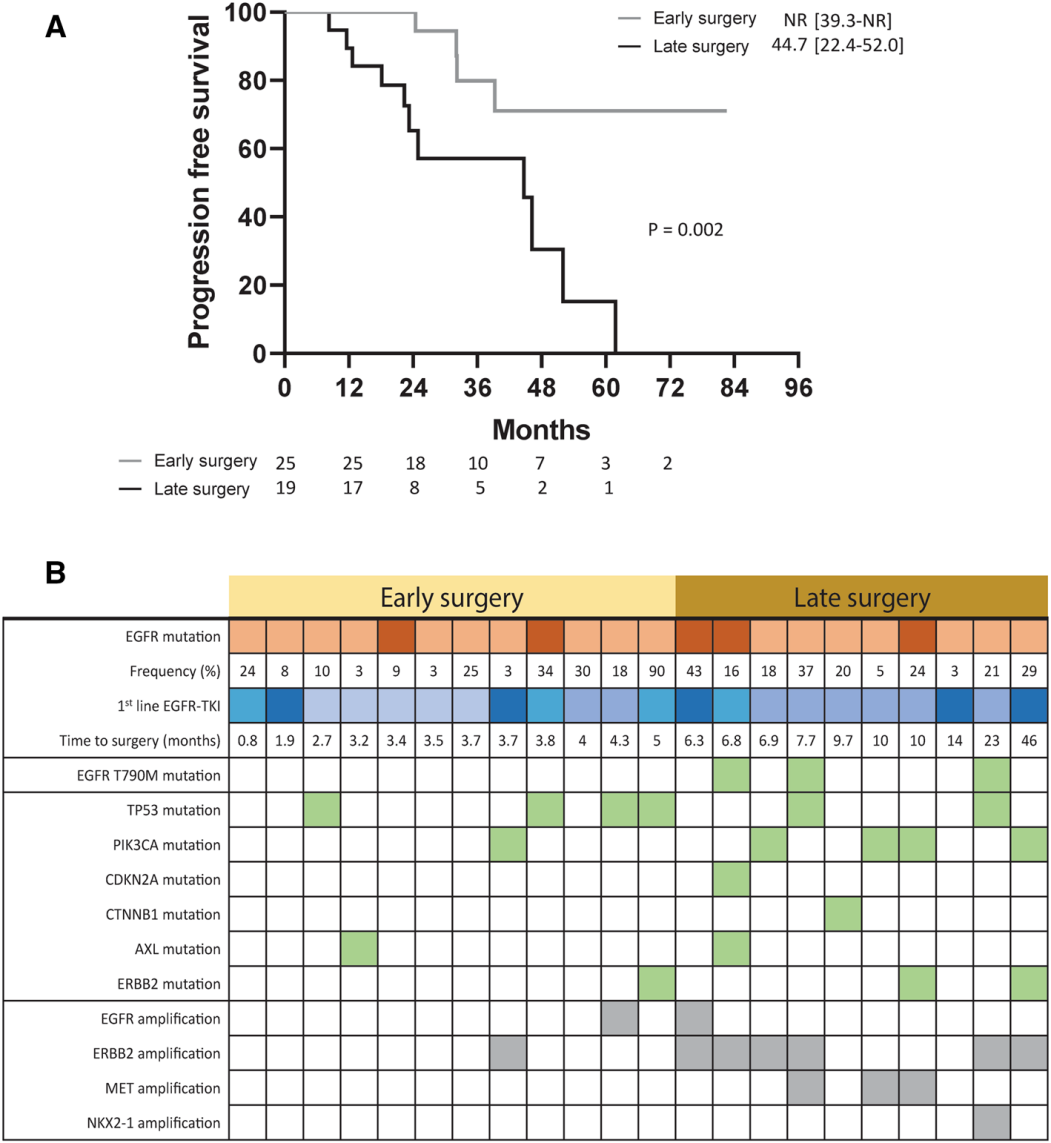
(**A**) Progression-free survival in patients who underwent early or late surgery after partial response to EGFR-TKI (**B**) Co-existing genomic alterations in patients within the early and late surgery groups.

Additionally, of the 44 patients who received surgery after partial response to EGFR-TKI treatment, 25 patients received surgery within six months of EGFR-TKI treatment initiation (early surgery group), while 19 patients received surgery after six months of EGFR-TKI treatment (late surgery group). The median PFS was not reached [interquartile range 39.3-NR months] among patients in the early surgery group, which was longer than patients in the late surgery group (Fig. 6A, p = 0.002).

### Resected tumor tissue genomic testing

Of the 44 patients who received surgery after partial response to EGFR-TKI, 22 had sufficient residual tissue for genomic testing, including 12 patients in the early surgery group and ten patients in the late surgery group (Fig. 6B). Three patients in the late surgery group had the T790M resistance mutation. In contrast, no patient in the early surgery group had that mutation. Moreover, all patients in the late surgery group harbored a co-existing mutation or amplification, known to decrease EGFR-TKI sensitivity, including TP53, PIK3CA, CDKN2A, CTNNB1, ERBB2, MET, and NKX2-1. In contrast, only six patients in the early surgery group harbored the co-existing mutations. More patients had co-existing mutations in the late surgery group than in the early surgery group (p = 0.009).

## Discussion

A previous study by Rusthoven et al. has demonstrated that the loco-regional progression was the predominant failure pattern among patients with advanced NSCLC^38^^.^ This result may indicate the potential role of local treatment to improve the first-line treatment efficacy. As a definite local treatment, surgical resection could also be considered a combination strategy to improve treatment outcomes in patients with advanced NSCLC. In the current study, we used propensity score matching to reduce the selection bias and found that the residual tumor resection after partial response to EGFR-TKI or primary tumor resection followed by EGFR-TKI had clinical benefits in both PFS and OS. Primary tumor resection was further confirmed as an independent and better prognostic factor using the Cox proportional hazards regression analysis (Table 2).

**Table 2:** Cox proportional hazards regression for progression-free survival and overall survival of all patients.

		Progression-free survival		Overall survival	
		HR (95% CI)	*p*-value	HR (95% CI)	*p*-value
Age	≥ 60 versus < 60	0.84 (0.50–1.39)	0.492	1.38 (0.68–2.80)	0.375
Sex	Male versus female	1.50 (0.85–2.66)	0.163	1.49 (0.69–3.24)	0.313
Tumor size	> 3 cm versus < 3 cm	1.33 (0.60–2.94)	0.489	1.49 (0.43–5.22)	0.533
Nodal involvement	Positive versus negative	2.06 (0.96–4.41)	0.063	1.42 (0.66–3.07)	0.377
EGFR mutation	Del 19 versus L858R	0.87 (0.50–1.53)	0.628	2.32 (0.95–5.70)	0.066
Stage	Newly diagnosed versus recurrence	0.71 (0.24–2.12)	0.545	0.77 (0.35–1.61)	0.276
Brain metastasis	Presence versus absence	2.14 (1.07–4.28)	0.032	1.15 (0.42–3.17)	0.785
EGFR-TKI	2nd generation versus 1st generation EGFR-TKI	0.57 (0.33–1.01)	0.052	0.56 (0.25–1.25)	0.156
Surgery	With primary tumor resection versus without primary tumor resection	0.19 (0.11–0.33)	< 0.001	0.14 (0.06–0.36)	< 0.001

*EGFR* epidermal growth factor receptor.

The study conducted by Chikaishi et al.^39^, which enrolled 38 patients with stage IV lung cancer, demonstrated that patients who underwent primary tumor resection as the first-line treatment had a 5-year overall survival rate of 29.0%, which was much higher than historically reported. Another study by Liu et al.^27^, which analyzed the SEER database, also suggested that combining thoracic surgery could improve the treatment efficacy of systemic chemotherapy. Other cohort studies conducted by Sun et al.^40^ and Chiang et al.^41^ also confirmed by multivariate analysis that surgical resection is an independent prognostic factor of improvement. However, all the studies mentioned above did not focus on patients harboring the EGFR mutation and the role of surgery in the treatment efficacy of EGFR-TKI. Recently, a retrospective study compared the treatment efficacy of EGFR-TKI with and without primary tumor resection. The study revealed that the significant benefit in PFS and OS could be achieved when combining primary tumor resection with EGFR-TKI. However, most patients in the study had recurrent NSCLC after previous curative surgery; the data from newly diagnosed NSCLC patients remains limited.

Moreover, the baseline characteristics between patients with and without primary tumor resection were imbalanced, which was insufficient to prove the clinical benefit^31^. In the current study, we used propensity score matching to adjust potential confounders. Further analysis of patients with newly diagnosed EGFR-mutant advanced NSCLC showed improved PFS and OS in the tumor resection group (Fig. 5). The Cox proportional hazards regression analysis also confirmed that surgery was an independent and better prognostic factor (Table 3).

**Table 3:** Cox proportional hazards regression for progression-free survival and overall survival of newly diagnosed patients.

		Progression-free survival		Overall survival	
		HR (95% CI)	p-value	HR (95% CI)	p-value
Age	≥ 60 versus < 60	0.71 (0.43–1.19)	0.195	1.09 (0.52–2.29)	0.811
Sex	Male versus female	1.57 (0.86–2.88)	0.141	1.48 (0.64–3.39)	0.359
Tumor size	> 3 cm versus < 3 cm	1.07 (0.47–2.46)	0.873	0.93 (0.26–3.30)	0.915
Nodal involvement	Positive versus negative	1.65 (0.75–3.60)	0.212	1.81 (0.52–6.30)	0.354
EGFR mutation	Del 19 versus L858R	0.79 (0.44–1.42)	0.428	1.19 (0.52–2.72)	0.678
Brain metastasis	Presence versus absence	2.25 (1.06–4.77)	0.034	1.13 (0.35–3.59)	0.843
EGFR-TKI	2nd generation versus 1st generation EGFR-TKI	0.63 (0.35–1.12)	0.113	0.62 (0.27–1.40)	0.250
Surgery	With primary tumor resection versus without primary tumor resection	0.14 (0.07–0.26)	< 0.001	0.12 (0.04–0.36)	< 0.001

*EGFR* epidermal growth factor receptor.

Instead of the EGFR T790M mutation, which is the well-known resistance mechanism after the use of first- or second-generation EGFR-TKI^42,43^, other co-existing genomic alterations have also been widely studied in patients with EGFR-mutant NSCLC received EGFR-TKI. A cohort study, which enrolled 16 patients with early-stage EGFR-mutant NSCLC, revealed that multiple truncal alterations, including TP53 mutations and loss of CDKN2A and RB1, were associated with high genomic instability and a higher proportion of co-existing genomic alterations^44^. A subsequent study enrolled 200 patients with metastatic EGFR-mutant NSCLC and further confirmed that co-existing genomic alterations, including ERBB2 and MET amplification, negatively affected the PFS in EGFR-TKI treatment^45^. According to the study conducted by Hata et al., the resistant mutation could emerge from pre-existing resistant clones or genetic evolution of EGFR-TKI tolerant cancer cells^46^. In the current study, all resected tumor specimens had preserved the original activating EGFR mutation. In addition, a higher proportion of co-existing mutations was found in patients within the late surgery group. These data implicated the presence of genetic evolution of drug-resistant cancer cells. Moreover, patients in the late surgery group had shorter PFS, resulting from the accumulation of resistant mutations. In summary, early surgical intervention after partial response to EGFR-TKI may be associated with a lower incidence of co-existing genomic alterations and could lead to a better treatment response to EGFR-TKI.

Previous studies had demonstrated that adjuvant chemotherapy could provide better disease-free survival among patients with early-stage NSCLC after surgery^47,48^. Recently, the phase 3 IMpower 010 study demonstrated that the implementation of atezolizumab could improve the disease-free survival of patients with early-stage NSCLC after surgery^49^. However, in the subgroup analysis, the presence of EGFR mutation would deteriorate the treatment efficacy. The possible explanation for the failure of immune checkpoint inhibitors may be secondary to the interaction between EGFR and programmed death ligand-1 (PD-L1). A previous study had demonstrated a significant correlation between the expression level of EGFR and PD-L1 from the analysis of the data from the Cancer Genome Atlas Program^52^. The expression of PD-L1 in EGFR-mutant NSCLC may result from the activation of the cell-intrinsic EGFR pathway instead of cell-extrinsic stimulation from the tumor immune microenvironment^53^.

After ADAURA study, the adjuvant osimertinib became the only FDA-approved therapy that could prolong disease-free survival in patients with stage IB to IIIA EGFR mutation-positive NSCLC^58^. However, the acquired resistance mechanism to adjuvant therapy also has an important role. In the final analysis of the ADJUVANT trial, only 36.8% of patients received subsequent targeted therapy in the gefitinib group, which is lower than those in the chemotherapy group^60^. This data implied that the EGFR-TKI-resistant tumor would occur after adjuvant-targeted therapy. The resistance mechanism to osimertinib is more complex, including on-target C797 mutation, activation of bypass pathway, and histological transformation^61^. Currently, there is no optimal subsequent therapy after acquired resistance to osimertinib. Furthermore, the use of osimertinib should also consider cardiotoxicity^62^, which is seldomly reported in first- or second-generation EGFR-TKIs. In the present study, we maintained systemic therapy using the same EGFR-TKI instead of switching patients to osimertinib, which may result in lower cardiac toxicity and a higher chance of allowing for subsequent targeted therapy. Our study had some limitations that need to be mentioned. First, this was a retrospective, single-institution study, and the number of patients in our cohort was limited. However, using the propensity score matching, we adjusted for patients’ demographic biases that are inevitable in real-world studies. Second, for correct matching, all the patients in the current study had a good performance status; whether the same result could be noted in patients with poor performance status remains unknown. Third, the late surgery group patients had shorter PFS and a higher proportion of co-existing genomic alterations, which implies early tumor resection may have more benefit. However, we did not perform NGS testing before the initiation of EGFR-TKI due to insufficient tissue samples. Whether early surgery could reduce the incidence of co-existing genomic alterations still warrants prospective study. Fourth, only two patients had tumor invasion to the mediastinum or chest wall in the surgery group. Although both patients had very good tumor response after EGFR-TKI therapy and post-operative N0 disease, the limited number of patients with tumor invasion to mediastinum or chest wall precludes a definitive conclusion. Whether residual tumor resection could provide clinical benefit in patients with tumor invasion to the mediastinum or chest wall needs further investigation. Fifth, although there is no significant difference in distant metastatic burden between patients with and without residual tumor resection, a marginally higher proportion of patients in the surgery group had bone metastasis. This result implies that the benefit of residual tumor resection may be limited to patients with a relatively low metastatic burden. A future prospective study is warranted to validate the result.

In conclusion, our study revealed that the combination of EGFR-TKI and tumor resection provided better PFS and OS than EGFR-TKI alone. Compared with a previous retrospective study^31^, we provided more evidence in patients with newly-diagnosed EGFR-mutant NSCLC. Moreover, we also found that the patients who underwent tumor resection within 6 months after the initiation of EGFR-TKI treatment had better PFS and a lower proportion of co-existing genomic alterations, which might imply the potential benefit of early surgical intervention. However, the use of tumor resection might be limited in patients with mediastinal invasion or a high metastatic burden because of limited patient number and unbalanced subgroup. A randomized phase III study comparing EGFR-TKI and surgery with EGFR-TKI alone is needed to verify whether the early tumor resection may increase survival.

## Supplementary Information

Supplementary Tables.

## Data Availability

The datasets generated during and/or analyzed during the current study are available from the corresponding author on reasonable request.
