# Supplementary material for: The surgical resection of the primary tumor increases survival in patients with EGFR-mutant advanced non-small cell lung cancer: a tertiary center cohort study
Source: Sci Rep. 2022 Dec 29;12:22560. doi: 10.1038/s41598-022-22957-9 (PMC9800377; doi:10.1038/s41598-022-22957-9)
Supplement: Supplementary file 1 — Supplementary Tables. [file 41598_2022_22957_MOESM1_ESM.docx]

Supplementary Table 1. The proportion of patients who received residual tumor resection in different subgroups

|  | Total population  (N=349) | Mediastinal lymphadenopathy  (N=294) | Brain metastasis  (N=96) | Liver metastasis  (N=39) | Bone metastasis  (N=80) | Adrenal metastasis  (N=24) |
| --- | --- | --- | --- | --- | --- | --- |
| Primary tumor resection  No primary tumor resection | 55 (15.8%)  294 (84.2%) | 44 (15.0%)  254 (85.0%) | 9 (9.4%)  87 (90.6%) | 2 (5.1%)  37 (94.9%) | 6 (7.5%)  74 (92.5%) | 0 (0.0%)  24 (100.0%) |

Supplementary Table 2. The metastatic burden of patients received primary tumor resection and patients received EGFR-TKIs alone

|  | Before propensity score matching | | | After propensity score matching | | |
| --- | --- | --- | --- | --- | --- | --- |
|  | Primary tumor resection (N=55) | No primary tumor resection (N=294) | P value | Primary tumor resection (N=53) | No primary tumor resection (N=53) | P value |
| Mediastinal lymphadenopathy  Distant metastasis  Brain  Liver  Bone  Adrenal gland | 44  9  2  6  0 | 254  87  37  74  24 | 0.218  0.044  0.053  0.021  0.028 | 42  9  2  6  0 | 45  9  5  13  1 | 0.447  1.000  0.241  0.076  0.315 |

Supplementary Table 3. The detailed surgical outcome in patients who received primary tumor resection

| Subject ID | T stage | Local invasion (mediastinum, chest wall or diaphragm) | Tumor location | Pre-OP lymph node stage | Post-OP lymph node stage | Surgical procedure | Undergone surgery by VATS | Resection margin |
| --- | --- | --- | --- | --- | --- | --- | --- | --- |
| 1 | T4 | No | RLL | 0 | 0 | Lobectomy | Yes | R0 |
| 2 | T2a | No | LLL | 0 | 0 | Segmentectomy | Yes | R0 |
| 3 | T4 | No | RLL | 2 | 0 | Segmentectomy | Yes | R0 |
| 4 | T3 | No | LLL | 1 | 1 | Lobectomy | Yes | R0 |
| 5 | T2a | No | RLL | 2 | 2 | Lobectomy | Yes | R0 |
| 6 | T2b | No | LLL | 0 | 0 | Lobectomy | Yes | R0 |
| 7 | T2b | No | RUL | 2 | 2 | Lobectomy | Yes | R0 |
| 8 | T1a | No | LUL | 3 | 0 | Segmentectomy | Yes | R0 |
| 9 | T2a | No | LUL | 3 | 2 | Lobectomy | Yes | R0 |
| 10 | T3 | No | LLL | 3 | 1 | Segmentectomy | Yes | R0 |
| 11 | T3 | No | RLL | 2 | 1 | Lobectomy | Yes | R0 |
| 12 | T4 | No | RLL | 2 | 0 | Segmentectomy | Yes | R0 |
| 13 | T2a | No | LUL | 2 | 0 | Lobectomy | Yes | R0 |
| 14 | T2a | No | RUL | 1 | 0 | Lobectomy | Yes | R0 |
| 15 | T1a | No | LUL | 3 | 0 | Segmentectomy | Yes | R0 |
| 16 | T2a | No | LUL | 0 | 0 | Segmentectomy | Yes | R0 |
| 17 | T4 | No | LUL | 2 | 1 | Lobectomy | Yes | R0 |
| 18 | T4 | No | RML | 3 | 0 | Lobectomy | Yes | R0 |
| 19 | T2b | No | RUL | 3 | 0 | Lobectomy | Yes | R0 |
| 20 | T3 | No | RUL | 2 | 0 | Lobectomy | Yes | R0 |
| 21 | T2b | No | LLL | 3 | 0 | Lobectomy | Yes | R0 |
| 22 | T2a | No | LLL | 2 | 0 | Segmentectomy | Yes | R0 |
| 23 | T4 | No | RLL | 2 | 0 | Lobectomy | Yes | R0 |
| 24 | T2a | No | LUL | 1 | 0 | Lobectomy | Yes | R0 |
| 25 | T3 | Yes | LLL | 3 | 0 | Lobectomy | Yes | R0 |
| 26 | T3 | No | LUL | 3 | 0 | Lobectomy | Yes | R0 |
| 27 | T3 | Yes | LUL | 3 | 2 | Lobectomy | Yes | R0 |
| 28 | T2a | No | LUL | 2 | 1 | Lobectomy | Yes | R0 |
| 29 | T2a | No | LLL | 0 | 0 | Lobectomy | Yes | R0 |
| 30 | T4 | No | RLL | 1 | 0 | Lobectomy | Yes | R0 |
| 31 | T2b | No | RUL | 3 | 1 | Lobectomy | Yes | R0 |
| 32 | T2a | No | LLL | 0 | 0 | Segmentectomy | Yes | R1 |
| 33 | T3 | No | RLL | 3 | 0 | Lobectomy | Yes | R0 |
| 34 | T4 | No | LUL | 0 | 0 | Segmentectomy | Yes | R1 |
| 35 | T4 | No | LLL | 3 | 0 | Lobectomy | Yes | R0 |
| 36 | T3 | No | RLL | 3 | 0 | Lobectomy | Yes | R0 |
| 37 | T3 | No | LUL | 0 | 0 | Lobectomy | Yes | R0 |
| 38 | T4 | No | RLL | 0 | 0 | Segmentectomy | Yes | R0 |
| 39 | T2b | No | LUL | 2 | 0 | Lobectomy | Yes | R0 |
| 40 | T4 | No | LLL | 0 | 0 | Lobectomy | Yes | R0 |
| 41 | T4 | No | LLL | 2 | 0 | Lobectomy | Yes | R0 |
| 42 | T4 | No | LLL | 3 | 0 | Lobectomy | Yes | R0 |
| 43 | T4 | No | RLL | 0 | 0 | Lobectomy | Yes | R0 |
| 44 | T3 | No | LUL | 1 | 0 | Segmentectomy | Yes | R1 |
| 45 | T4 | No | RLL | 2 | 0 | Segmentectomy | Yes | R0 |
| 46 | T3 | No | LLL | 3 | 0 | Segmentectomy | Yes | R0 |
| 47 | T3 | No | RLL | 3 | 2 | Lobectomy | Yes | R0 |
| 48 | T2b | No | LUL | 2 | 2 | Lobectomy | Yes | R0 |
| 49 | T4 | No | RLL | 2 | 2 | Lobectomy | Yes | R0 |
| 50 | T2b | No | RUL | 3 | 1 | Lobectomy | Yes | R0 |
| 51 | T3 | No | RUL & RML | 3 | 1 | Lobectomy | Yes | R0 |
| 52 | T3 | No | LUL | 3 | 0 | Lobectomy | Yes | R0 |
| 53 | T4 | No | RML | 3 | 0 | Segmentectomy | Yes | R0 |

LLL, left lower lung; LUL; left upper lung; RLL, right lower lung; RML, right middle lung; RUL, right upper lung.

Supplementary Table 4. The pathological response in patients who received primary tumor resection after partial response to EGFR-TKI

| Subject ID | Pre-treatment tumor size (cm) | Post-treatment tumor size (cm) | Pre-OP lymph node stage | Post-OP lymph node stage | Pathological response | Post-operative complication |
| --- | --- | --- | --- | --- | --- | --- |
| 1 | 4.8 | 4.0 | 0 | 0 | 5% | No |
| 3 | 2.5 | 2.0 | 2 | 0 | 10% | Dyspnea |
| 4 | 5.4 | 2.6 | 1 | 1 | 20% | Dyspnea |
| 11 | 4.8 | 3.4 | 2 | 1 | 90% | No |
| 12 | 3.5 | 2.1 | 2 | 0 | 95% | wound infection |
| 13 | 3.2 | 3.0 | 2 | 0 | 95% | No |
| 14 | 4.0 | 3.8 | 1 | 0 | 10% | No |
| 15 | 1.6 | 1.5 | 3 | 0 | 95% | No |
| 16 | 2.2 | 2.0 | 0 | 0 | 5% | No |
| 17 | 3.8 | 2.7 | 2 | 1 | 60% | No |
| 18 | 3.1 | 2.7 | 3 | 0 | 80% | No |
| 19 | 4.2 | 2.6 | 3 | 0 | 60% | No |
| 20 | 6.4 | 5.3 | 2 | 0 | 95% | No |
| 21 | 4.4 | 2.9 | 3 | 0 | 98% | No |
| 22 | 3.2 | 2.9 | 2 | 0 | 5% | No |
| 23 | 2.4 | 1.8 | 2 | 0 | 5% | Prolonged air leak |
| 24 | 1.8 | 1.6 | 1 | 0 | 95% | No |
| 25 | 3.5 | 2.6 | 3 | 0 | 50% | No |
| 26 | 4.3 | 2.9 | 3 | 0 | 30% | No |
| 27 | 3.7 | 1.8 | 3 | 2 | 5% | Prolonged air leak |
| 29 | 3.3 | 2.8 | 0 | 0 | 40% | No |
| 30 | 9.8 | 5.6 | 1 | 0 | 100% | No |
| 31 | 3.2 | 2.9 | 3 | 1 | 70% | No |
| 32 | 3.7 | 2.3 | 0 | 0 | 10% | No |
| 33 | 7.0 | 5.3 | 3 | 0 | 97% | No |
| 34 | 3.5 | 3.8 | 0 | 0 | 20% | No |
| 35 | 6.5 | 3.4 | 3 | 0 | 93% | No |
| 36 | 6.8 | 3.7 | 3 | 0 | 60% | No |
| 37 | 6.4 | 3.0 | 0 | 0 | 5% | No |
| 38 | 5.4 | 5.4 | 0 | 0 | 5% | No |
| 40 | 3.6 | 2.7 | 0 | 0 | 5% | No |
| 41 | 2.4 | 1.9 | 2 | 0 | 95% | No |
| 42 | 4.1 | 2.4 | 3 | 0 | 40% | No |
| 43 | 4.2 | 2.5 | 0 | 0 | 90% | No |
| 44 | 3.6 | 1.2 | 1 | 0 | 80% | No |
| 45 | 2.6 | 2.1 | 2 | 0 | 80% | No |
| 47 | 3.6 | 3.0 | 3 | 2 | 70% | No |
| 48 | 4.0 | 3.2 | 2 | 2 | 20% | No |
| 50 | 5.0 | 3.0 | 3 | 1 | 50% | No |
| 51 | 4.1 | 1.8 | 3 | 1 | 70% | No |
| 52 | 3.4 | 3.1 | 3 | 0 | 80% | No |
| 53 | 6.5 | 3.6 | 3 | 0 | 10% | No |
